# Effect of Ultrasound Standing Wave-Induced Acoustophoresis in Monoglyceride Oleogel Structuration

**DOI:** 10.1021/acs.cgd.5c00291

**Published:** 2025-06-03

**Authors:** Petri Lassila, Thomas Zinn, Jere Hyvönen, Enriqueta Noriega Benitez, Paavo Penttilä, Ari Salmi, Fabio Valoppi

**Affiliations:** † Electronics Research Laboratory, Department of Physics, 3835 University of Helsinki, P.O. Box 64 (Gustaf Hällströmin katu 2), FI-00014 Helsinki, Finland; ‡ 120796 Diamond Light Source Ltd., Didcot, Oxfordshire OX11 ODE, United Kingdom; § Department of Bioproducts and Biosystems, School of Chemical Engineering, 174277 Aalto University, Vuorimiehentie 1, Espoo 02150, Finland; ∥ Department of Food and Nutrition, University of Helsinki, P.O. Box 66 (Agnes Sjöbergin katu 2), FI-00014 Helsinki, Finland; ⊥ Helsinki Institute of Sustainability Science, Faculty of Agriculture and Forestry, University of Helsinki, FI-00014 Helsinki, Finland; # Helsinki Institute of Life Science, University of Helsinki, 00014 Helsinki, Finland; ∇ Perfat Technologies, Agnes Sjöbergin katu 2, 00790 Helsinki, Finland

## Abstract

Ultrasound standing waves (USW) produce a force capable of displacing micrometer-sized free-flowing particles in a fluid, wherein this phenomenon is also referred to as acoustophoresis. However, the effect of acoustophoresis on dynamically changing and growing crystal networks is unclear. An example of such a system are monoglyceride (MG)-based oleogels, which are free-flowing lipids (e.g., vegetable oils) structured with a lipid-crystal network. In this work, we use MG oleogels as an example system to investigate the acoustophoretic effect on the structuration of a growing crystal network. For this purpose, multifaceted characterization is conducted utilizing optical and coded excitation scanning acoustic microscopy as well as small-angle X-ray scattering, respectively. The optical microscopy results show that USW produces local density differences of the structuring crystalline material and induces the orientation of the MG platelets. X-ray diffraction measurements confirm these findings and show a 23% average increase in MG platelet correlation length, which can be linked to platelet thickness, as well as an increase in the MG nanoplatelet surface smoothness. These findings produce a foundation for better understanding the effect of acoustophoresis in dynamically developing lipid-based materials and illuminate the mechanical changes that arise because of USW treatment.

## Graphical abstract

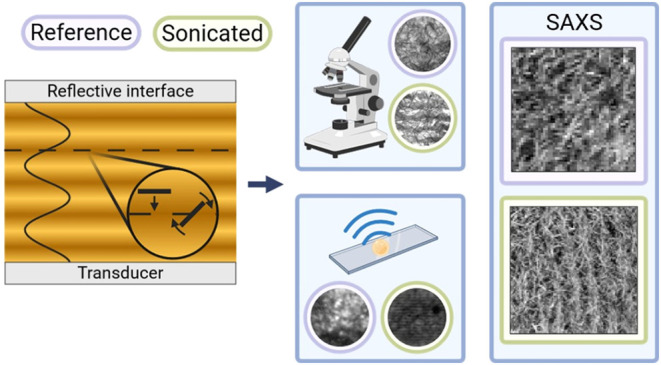

## 1 Introduction

Unhealthy diets continue to be a major driver in the onset of chronic diseases relating to obesity in the developed world, such as cardiovascular diseases, type 2 diabetes, and metabolic syndrome. Overconsumption of saturated fatty acids is a contributor in this regard.^1,2^ However, saturated fats have proven to be difficult to replace due to their desirable sensorial and functional properties. As such, a considerable amount of research has been done to further the process of gelling free-flowing oils rich in unsaturated fatty acids, i.e., oleogels, over the past decades.^3^ Oleogels are applied with the aim of replacing saturated fats, aiming to maintain similar mechanical and sensorial properties as traditional solid fats (e.g., butter and palm oil-based fats) but reducing or eliminating the total saturated fat content. The mechanism of oleogelation involves trapping of the lipid fraction in a solid matrix network formed from structuring agents. Various materials can be used as structuring agents (polysaccharides, proteins, or low-molecular-weight self-assembling molecules) depending on the method of oleogelation.^4,5^ However, one of the simplest and straightforward approaches is to use a direct, thermal cycle method.^6^ Typically, this entails heating and mixing a mixture of gelators in vegetable oil until solubilization is achieved. The solution then experiences cooling, wherein the solid fraction starts to crystallize, eventually forming a network of lipid crystals entrapping the oil. The mechanical properties of the formed matrix are highly dependent on the nucleation and growth dynamics of the forming lipid crystals. These processes can commonly be altered by controlling the rate of cooling, or by applying shear forces during crystal formation.^7–9^ However, the state-of-the-art methods do not provide avenues for precise control of the final formed lipid-crystal network, resulting in an inefficient design and underutilization of the solid fraction.

It is well known that engineered stress-resistant structures are tailorable to obtain desirable mechanical behavior such as increased stiffness. For example, various cellular structures, such as the hexagonal honeycomb structure, offer an increased stiffness-to-density ratio when compared to a fully solid alternative.^10^ It is the focus of our research group to engineer the lipid-crystal network of oleogels to obtain more desirable mechanical behavior by spatially organizing the microcrystals during their formation. To this aim, we explored the use of acoustophoresis, that is, the phenomena of particle migration in a fluid through interaction with an acoustic wave. An acoustic wave exerts a force, the acoustic radiation force, on particles suspended in a fluid capable of displacing the particles on the basis of the properties of the acoustic field. Commonly, ultrasonic standing waves (USW) are utilized in particle manipulation because they provide sufficiently small wavelengths (submillimeter) through high frequencies (single-digit MHz), and a predictable and efficient particle movement. Particles in a USW field will agglomerate in the nodal- or antinodal planes of the standing wave depending on their mechanical properties in relation to the suspending fluid, as well as orient along the planar directions.^11–14^ In relation to conventional methods employed in lipid-crystal control (application of shear, control of cooling rate, and changing formulation), USW structuring can potentially provide a high-resolution, large-scale, and time-efficient approach to lipid-crystal structuration due to the volumetric capabilities of acoustic fields.

Our research group has previously demonstrated the feasibility of applying USW structuring on a direct, thermal cycle formed oleogel.^15,16^ We showed the capability of forming submillimeter structures in a volume of over 100 mL of monoglyceride (MG) oleogel, without significantly diverging from reference production times. We also demonstrated that USW-treated oleogels showed a change in their mechanical properties as measured by uniaxial compression, demonstrating that the structural changes produced by ultrasound induced intrinsic mechanical functionality. The objective of the study presented here is to obtain a deeper understanding of the morphological changes produced by USW treatment, which led to the mechanical changes seen in monoglyceride oleogels. For this purpose, bright-field (BF) optical microscopy, coded excitation scanning acoustic microscopy (CESAM), and small-angle X-ray scattering (SAXS) are utilized to produce a comprehensive view into the microstructure of USW-treated oleogels. The results obtained in this work prove the effects of USW at the micro- and nanostructural levels in monoglyceride-based oleogels, enabling the further development of USW-structured lipid-crystal oleogels.

## 2 Materials and Methods

### 2.1 Materials

Rapeseed oil was purchased from a local grocery store (Keiju rypsiöljy, Bunge Finland Oy, Raisio, Finland). Myverol 18-04K saturated monoglyceride (MG) (fatty acid composition 1.4% C14:0, 59.8% C16:0, 38.8% C18:0; melting point 68.0 ± 0.5 °C) was donated by Kerry Ingredients and Flavor (Bristol, U.K.).

### 2.2 Sample Preparation

Oleogels were prepared by mixing 5 or 10% MG in rapeseed oil. The mixtures were heated under continuous stirring to 80 °C and kept at the target temperature for at least 15 min. The mixture was then poured into a custom-built ultrasonic treatment chamber. During cooling, samples were subjected to low-intensity ultrasonic standing waves, with a pressure amplitude of the standing wave of 120 kPa.^15^ USW treatment was applied at a frequency of 2.25 MHz. Samples were allowed to rest overnight in the chamber before removal. Samples were sliced utilizing a razor blade, mounted in brass holders provided by Diamond Light Source, and shipped for SAXS measurements. A similar slicing procedure was used to prepare samples for optical microscopy and coded excitation scanning acoustic microscopy. All concentrations are reported as % (w/w) unless otherwise specified. Production of reference samples was performed using the same procedure, without the excitation of ultrasonic waves.

### 2.3 Bright-Field Microscopy

Reference and USW-treated sample microstructures were visualized using a Zeiss Axio Lab A1 optical bright-field microscope (Oberkochen, Germany) connected to a Zeiss Axiocam 305 color camera. Samples were visualized at room temperature by using a 5× objective. Images were acquired and processed using application software ZEN 2.6 (Zeiss).

### 2.4 Small-Angle X-ray Scattering

Micro- and nanostructural investigations on USW-treated and reference oleogels were conducted utilizing small-angle X-ray scattering. Measurements were performed at the Diamond Light Source synchrotron (Harwell Science and Innovation Campus, Oxfordshire, U.K.), beamline I22. A 17 μm (*h*) × 23 μm (*v*) X-ray beam was used to perform a raster scan of 2 mm × 2 mm for sonicated samples and an area of 1 mm × 1 mm for reference samples. The step size for the scan was 20 μm, resulting in 101 data points for sonicated and 51 data points for reference samples. The photon energy of the X-ray beam was 14 keV. A silicon drift detector (Pilatus P3-2M, DECTRIS Ltd., Baden, Switzerland) was placed 3.15 m from the sample. The scattering data was reduced into 2D images of 1253 × 1253 pixels, with the magnitude of the scattering vector *q* and the azimuthal angle χ as the axes. The magnitude of the scattering vector is defined as *q* = (4π/λ) sin­(θ/2), where θ is the scattering angle and λ is the X-ray wavelength. Data processing was carried out using DAWN 2.32 (https://dawnsci.org/) and custom-written Python scripts.

### 2.5 Coded Excitation Scanning Acoustic Microscopy

A coded excitation scanning acoustic microscope (CESAM)^17^ was used to image the local acoustic attenuation in both USW-treated and reference-sliced Oleogel samples. The device employs coded excitation for increased signal-to-noise ratio. For this application, we used a spherically focusing 30 MHz immersion transducer (Panametrics V375, 30/25, *F* = 0.75″ OLF, from Baker Hughes, Houston, TX, USA). The frequency range (8–30 MHz) was chosen as a compromise between penetration depth, lateral resolution, and axial resolution.

Several 2-mm-thick samples were prepared and then placed on 200-μm-thick 22 mm × 22 mm glass microscope slides. No second coverslip was used, and one face of the gel remained uncovered. The sample was mapped with a 2D raster scanned grid. All measurements were performed in deionized, degassed, room-temperature water.

### 2.6 Data Analysis

All determinations are expressed as mean ± standard deviation, unless otherwise specified. Data was visualized utilizing MATLAB R2022a (MathWorks, Inc., Natick, Massachusetts, USA) and packages MagInset,^18^ in combination with Coord2norm.^19^

#### 2.6.1 SAXS Orientation Analysis

To produce radial scattering intensity plots from the 2D images, the images were averaged over azimuthal angle χ. Then, the interplanar distance *d* was calculated from the diffraction peak position *q*_peak_ at *q* = 0.13 Å^–1^ using Bragg’s law *d* = 2π/*q*_peak_. The interplanar distance *d* was utilized to estimate length scales observed in SAXS data.^20^ In addition, for platelet-like crystals, the full width at half-maximum (fwhm) of the diffraction peak can provide an estimate of the correlation length (i.e., thickness) of the nanocrystals through the Scherrer equation ξ = 2π/Δ*q*,^20^ where Δ*q* is the fwhm of the diffraction peak, obtained from the Debye–Scherrer ring. SciPy (version 1.14.0) peak finding algorithm, assuming a Gaussian peak shape, was used to determine the main diffraction peak location and fwhm.

A power-law equation^21,22^ was utilized to evaluate the nanoscale structure of the MG crystals in the linear region observed at low *q* in a double-logarithmic plot.

1 $$I\left(q\right)=\frac{B}{q^{P}}$$

where *P* is the Porod exponent and *B* is a fitted constant.

To analyze the orientation of the crystals, the 2D scattering images were averaged over the *q* range 0.11–0.15 Å^–1^ to obtain intensity distributions over azimuthal angle χ, which represent the orientation of the crystals producing the peak at *q* = 0.13 Å^–1^. To enhance the clarity of these intensity profiles, a convolution operation with a kernel size of 30 was applied, smoothing the data, and making the distributions more interpretable. Hermans orientation function is a measure of orientation, commonly used to measure the chain orientation of polymers in optical birefringence measurements, as well as X-ray diffraction and infrared spectroscopy.^23^ From the Hermans orientation function, we can obtain an orientation factor (OF) from the second spherical harmonic of the Hermans orientation function *f*(χ):

$$f_{2}\left(\chi \right)=\frac{1}{2}\left(3\left\langle \cos^{2}\left(\chi \right)\right\rangle -1\right)$$

Here, χ is the angle between the longitudinal axis of the MG platelets and the reference direction. Then, the term ⟨cos^2^(χ)⟩ can be approximated:^24^

$$\left\langle \cos^{2}\left(\chi \right)\right\rangle =\frac{\int_{0}^{\pi /2}I\left(\chi \right)\sin\left(\chi \right)\cos^{2}\left(\chi \right)d\chi }{\int_{0}^{\pi /2}I\left(\chi \right)\sin\left(\chi \right)d\chi }$$

where *I*(χ) is the scattered intensity as a function of the azimuthal angle. The OF assumes values between −0.5 and 1, wherein a value of −0.5 indicates orientation of the plane normal of the lipid-crystal platelet perpendicular to the director, a value of 0 denotes isotropic orientation, and a value of 1 shows orientation completely parallel to the director. The term director is used to indicate the 0° direction in relation to the scanning axis. It should be noted that since in our samples any orientation in the imaged samples is not necessarily uniaxial, as the Hermans orientation function assumes, the OF should be considered as a simple estimate of the true orientation of lipid crystals in the sample.

#### 2.6.2 Microscopy Image Analysis

Captured optical microscopy images were subjected to 2D FFT image analysis. The methodology outlined here is a modified approach shown by Ayres et al.,^25^ with additional bandpass filters used in the spatial frequency space. Image analysis was performed in MATLAB R2022a, with functions available in the Image Manipulation Toolbox (MIMT). The image processing consisted of five steps: (i) microscopy images (stored as uncompressed.TIF files) were imported into MATLAB (Figure 1, step i); (ii) the complement of the imported image was taken, and a circular diffuse (Gaussian) mask was applied (Figure 1, step ii); (iii) following this, the 2D FFT operation was numerically carried out utilizing the MATLAB’ built-in fft2­() function, and the result was centrally shifted using the fftshift­() function (Figure 1, step iii); (iv) sharp bandpass filters were applied once again utilizing MIMT (Figure 1, step iv), where the appropriate spatial frequency axes were calculated utilizing the spatial resolution provided by our microscope; and (v) the resulting spatial frequency space matrix was mapped to polar coordinates, with the origin at the center of the matrix (Figure 1, step v). The integration over the polar angle was conducted through rounding of the polar angle to the nearest integer and summing all elements within the angle. The resulting power spectra were plotted using a polar plot (Figure 1). The obtained angular dependence was further smoothed using the MATLAB’ inbuilt Savitzky-Golay filter to ease comparison between different samples.

**Figure 1:**
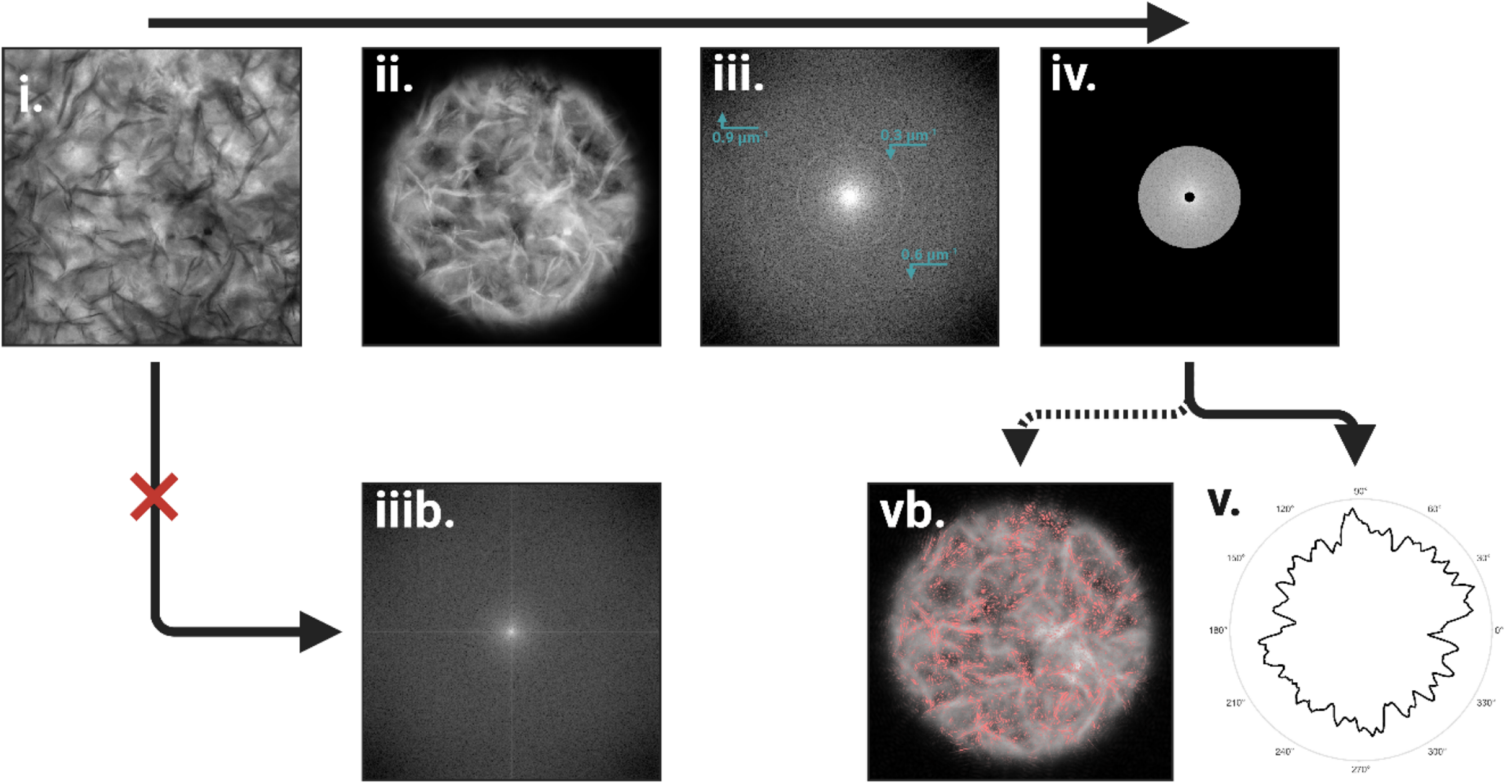
(i–v) Sequential image analysis steps for producing power spectral data. (i) Imported microscope image of sample. (ii) Taking the negative of the original image and application of a Gaussian mask to the image. (iii) 2D FFT image where Gaussian mask has been applied, showing remaining circular artifacts in cyan. (iiib) 2D FFT image where no Gaussian mask has been applied. (iv) Application of a bandpass filter to the observed power spectral distribution. (v) Integration over the polar angle to obtain the angular dependence of the power spectral distribution. (vb) Alternative manual confirmation of the extraction of desired elements in the original image through the inverse Fourier transform. Arrows indicate the sequential order of steps.

## 3 Results and Discussion

### 3.1 Optical Microscopy-Based Orientation Analysis

Bright-field (BF) microscopy images of MG oleogels (R, reference; S, sonicated) are shown in Figure 2. In images taken of sonicated samples, a band structure of dark and light regions is visible, indicating regions of denser and sparser material. This structure corresponds to the nodal planes of the applied USW, as expected. On the other hand, in the image taken of the reference sample, no such consistent variation can be seen. Images of both samples show, in addition to this, individual MG crystals which constitute the lipid-crystal network. It is expected that elements closer to the imaging side (camera) will appear sharper and in more detail in transmission microscopy. While elements closer to the backlight will appear blurrier due to passing through the material, explaining the appearance of singular sharp crystal and a blurred mass of crystals in the microscopy images. Comparing 10 and 5% images, it can be observed that single platelets in 10% appear in fewer number and are longer. There does not appear to be significant changes in platelet size and number in between reference and sonicated samples (for both 10 and 5%). Any preferential orientation for the individual crystals is difficult to distinguish based on qualitative interpretation of the BF microscopy images alone due to their blurriness. For this purpose, image analysis was carried out.

**Figure 2:**
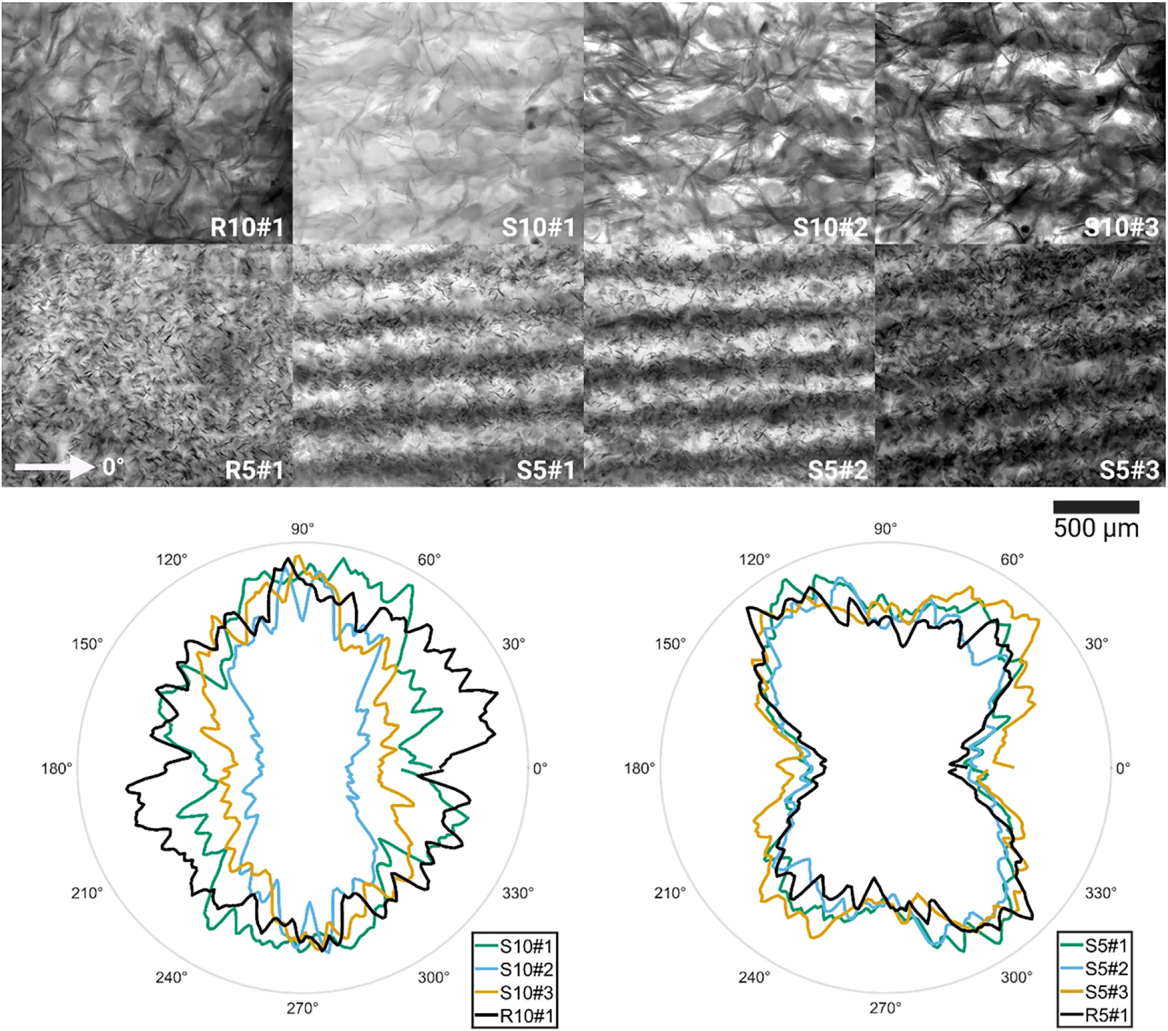
Top two rows: Bright-field optical microscopy images of 10% (S10#) and 5% (S5#) MG sonicated samples from distinct locations. Left-most column: bright-field optical microscopy images of 10% (R10#1) and 5% (R5#1) MG reference samples. Lowest row: Power spectra plots for 10% (left) and 5% (right) MG samples, respectively.

The image analysis process was conducted using a bandpass filter of 0.025–0.25 μm^–1^, corresponding to element sizes in between 4 and 40 μm in thickness. The process of image analysis is highlighted in Figure 1. The image analysis consists of taking the 2D FFT of the images under processing, followed by taking the power spectra of the Fourier domain image by squaring the image and normalizing. This image is then integrated over the radius, with the origin at the center of the image and obtaining polar angle dependence. For the purposes of our analysis, applying the Gaussian mask and bandpass filter (Figure 1ii,iv) is of significant importance. The effect of not applying a Gaussian mask on the original image can be seen in Figure 1iiib, wherein intense vertical and horizontal lines can be observed. As these lines are intense, observing any differences in orientation becomes challenging. Applying the Gaussian mask removes these artifacts produced by the image edges but introduces artifacts with a circular shape (Figure 1, cf. step iii with iiib); these are however easier to eliminate from the analysis. In Figure 1iii, three ring artifacts are indicated with arrows, with the first appearing at a radius of 0.3 μm^–1^ and the second at 0.6 μm^–1^, and third ring artifact at 0.9 μm^–1^. While the first and second ring artifacts show a uniform intensity distribution over the polar angle, the third ring can be seen to show aliasing (bending of the circle near the corners), which results in an uneven angular dependence of the intensity. This can make interpreting slight changes in preferential orientation difficult, so a low-pass filter is applied in step iv to exclude these artifacts from the analysis. Additionally, a high-pass filter is also applied to avoid issues arising from poor resolution near the origin. This issue can be mitigated by introducing zero-padding in step ii, but for the purposes of our analysis, this was not deemed necessary. Manual verification of proper filtering was performed by inverse Fourier transform, an example of which can be seen in Figure 1, step vb.

For 10% MG sonicated samples (Figure 2 S10#), all images showed a clear preferential direction, as can be observed in the integrated power spectra polar plot (Figure 2). However, the observed degree of preferential orientation (i.e., degree of ellipsoidal shape) varied based on the sampled location. Among 10% MG sonicated samples, image S10#2 showed the highest degree of orientation wherein the power spectra (Figure 2, left power spectra, cyan line) adopted a highly ellipsoidal/rectangular shape toward 90° (and symmetrically 270°), corresponding to the direction of the *y*-axis in Figure 2 S10#. Image S10#3 showed the second highest degree of orientation, and S10#1 the least when comparing the sonicated sample images. For the 10% MG reference sample image (Figure 2 R10#1), a more oval/rectangular shape could be observed. Notably, a dip in the degree of orientation can be seen at 0° (and 180°) for image R10#1.

The image analysis was repeated for 5% MG reference and sonicated samples (Figure 2, R and S5#), with the same parameters as for 10% MG. As with 10% MG samples, a repeating band-like structure (consisting of numerous platelets) is visible for 5% MG sonicated sample BF images (Figure 2, S5#). And as expected, no repeating structure is visible for the reference case (Figure 2 R5#1). It can be generally observed from the BF images that single platelets in the 5% MG case are smaller than for 10% MG samples. This appears to be the case regardless of whether sonication was applied. The results of the image analysis can be observed in Figure 2, right power spectra. Unlike the 10% MG case, the application of sonication does not seem to affect the preferential orientation of 5% MG samples. All images for 5% MG show preferential orientation toward approximately 55° (symmetrically 235°) and 130° (symmetrically 180°). This is assumed to be an artifact produced by the sampling process, wherein samples were compressed before imaging.

Overall, these findings are in line with previous observations.^15^ The effect of sonication was to increase the density of crystalline material in the nodal planes, the distance between the bands being approximately 0.3 mm, corresponding to the used ultrasound wavelength (approximately 0.65 mm), as expected. However, optical microscopy allowed for the identification of individual crystals closer to the sample surface facing the camera. While these crystals are suspected to be affected by sample handling (cutting and squeezing), overall, they provide an outlook into the effect of USW on Oleogel structuration. An important finding was that the application of USW produced orientation of the individual crystals parallel to the band structure for 10% MG sample, as it can be observed from the power spectra in Figure 2, left. This is generally in line with what is expected for free elongated particles,^13,14^ but a similar observation has not been done for materials dynamically forming a continuous supporting network. Interestingly, there was a varying degree of observed orientation between images captured from different regions. This is explainable either as artifacts produced by sample handling, local inhomogeneities in MG concentration affecting the structuration, or by an unideal acoustic field utilized in the USW treatment. As was detailed in a previous publication,^15^ the piezoceramic used for the treatment is a circular pz26 ceramic, with a diameter of 50 mm. For such a transducer, the near-field distance can be calculated to be approximately 1 m at 2.25 MHz.^26^ Though multiple acoustic reflections in the sonicated material exist to produce the standing wave, the existence of local pressure maxima and minima cannot be ruled out. Such inhomogeneities in the acoustic pressure field would result in regions in which the applied acoustic radiation force would be small or nonexistent. As such, the utilized transducer can be a contributing factor to the local variations in the obtained orientation. Unlike 10% MG, 5% MG sonicated samples showed no change in the preferred orientation in comparison to the reference case, while changes in the local density of crystalline material were obtained, as with 10% MG. 5% MG oleogels are generally softer, indicating a weaker crystalline network. As such, it cannot be ruled out that the lack of orientation for sonicated 5% MG samples would have been entirely due to artifacts produced by sample handling (cutting, squeezing). However, it is visibly apparent that the sizes (length and diameter) of individual crystals in the 10% MG and 5% MG cases (cf. Figure 2 R/S5# with Figure 2 R/S5#) are different.

### 3.2 CESAM Imaging of Reference and USW-Treated Oleogels

CESAM is an imaging method that is sensitive to the local variations in mechanical properties of the imaged sample. We utilized CESAM in a pulse-echo configuration to capture information about the internal structure of 10 and 5% MG samples. These results can be seen in Figure 3, where lighter areas in the images indicate regions where less attenuation has occurred, and darker regions indicate regions in the reverse. As discussed in a previous publication, wherein we fitted CESAM data with an empirical frequency-dependent power-law,^15^ this local variation in attenuation is likely due to acoustic scattering from the MG platelets. This leads to the interpretation that darker areas are regions with higher MG concentrations and lighter areas regions with lower concentrations.

**Figure 3:**
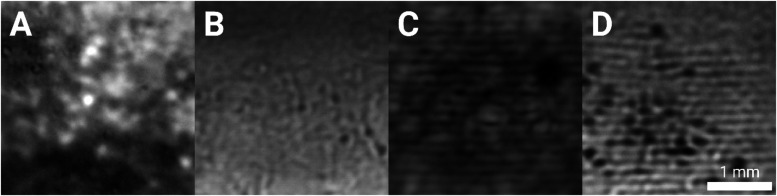
Coded excitation scanning acoustic microscopy images for (A) 10% MG reference sample, (B) 5% MG reference sample, (C) 10% MG sonicated sample, and (D) 5% MG sonicated sample. Images are 3 × 3 mm^2^ in size. The scale bar shown applies for all images (A–D).

Figure 3A,C shows CESAM images for 10% MG oleogels for both reference and sonicated cases, respectively. Both cases show local variation in the density of crystalline material. This natural inhomogeneity is due to local variations in concentration and can also be observed in the 5% MG samples (Figure 3B,D). However, comparing 10% with 5%, a higher degree of clumping (inhomogeneity) can be observed in both the reference and sonicated cases. The sonicated samples (Figure 3C,D) show, in addition to this inhomogeneity, an organization of the crystalline material into repeating lines running horizontally in the images. Interestingly, comparing the 5% reference and sonicated samples (cf. Figure 3B,D) more aggregation can be observed in the sonicated sample, especially around the bands of localized crystalline material, which corresponds to our previous CESAM findings.^15^

Scanning acoustic microscopes measure the local mechanical properties of samples by using focused ultrasonic beams. Compared to optical microscopes, where the contrast arises from the interaction of electromagnetic waves with the internal structure of the samples, the acoustic contrast is due to a mechanical wave interaction. This interaction can take place at the sample interfaces or within the sample due to local variation in mechanical properties. With MG oleogels, this is intrinsically linked to the concentration of crystalline material, and the following localized scattering of ultrasonic waves (wavelength 50–190 μm).^15^ Due to the presence of acoustical interfaces (crystal–oil interface) around the same physical dimension as the wavelength, strong scattering is present, leading to the contrast seen in the images. CESAM thus gives an indication of local density differences. This was observed in Figure 3, where the influence of USW treatment led to band-like denser and sparser regions of MG concentration. The observations made here agree with the obtained optical microscopy data. As the contrast mechanism is mechanical by nature, this shows that USW treatment had a tangible effect on the local mechanical properties.

### 3.3 SAXS Analysis of Reference and USW-Treated Oleogels

SAXS measurements were carried out on sliced 10 and 5%, reference, and sonicated samples to obtain information on the local orientation distribution of MG crystalline platelets in the formed oleogel. Example raw SAXS images can also be found in the Supporting Information (Figure S1). Figure 4A shows the radial intensity as a function of the magnitude of the scattering vector *q* (averaged over the scanned area), as averaged over the integrated diffraction pattern, capturing both low-*q* (below 0.1 Å^–1^) and high-*q* (above 0.1 Å^–1^) SAXS regimes. There are three notable regions in the radial plot present in all samples. Between *q-*values of 0.005 and 0.04 Å^–1^, we can observe the power-law region, wherein the diffracted intensity follows an empirical model (eq 1). Following the power-law region, a shoulder can be observed between *q* values of 0.04–0.08 Å^–1^. This is presumed to be a consequence of the Kapton walls used in the holder and as such provides no useful information on the samples. Finally, the main diffraction peak, from the lamellar structure of the MG crystals,^27,28^ is observed at around *q* = 0.13 Å^–1^. Comparing the 5 and 10% MG curves in Figure 4A, a slight increase in the maximum intensity at *q* = 0.13 Å^–1^ can be noticed in the case of sonicated samples (Figure 4A, inset). In addition, 10% MG oleogel samples exhibit a generally larger intensity when compared with 5% MG oleogel samples. This is due to the 10% sample having a higher concentration of crystalline material, which means there are more lamellae that scatter, leading to a higher intensity.

**Figure 4:**
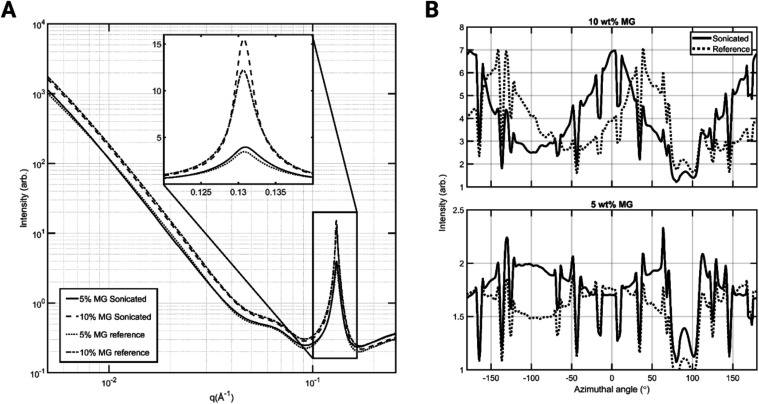
(A) Intensity as a function of *q* (integrated over azimuthal angle) with an inset showing the diffraction pattern in detail around the main diffraction peak. (B) Intensity as a function of the azimuthal angle at *q* = 0.13 Å^–1^. (A) and (B) are averages over the scan area of 101 × 101 points for sonicated samples, and 51 × 51 points for reference samples.

Adjacent to the radial plots, in Figure 4B are the azimuthal intensity plots averaged over the scanned area at *q* = 0.13 Å^–1^. In the azimuthal plots, a higher intensity is reflective of a higher prevalence of MG platelet orientation to that direction (i.e., where the normal of the platelet face is oriented). The azimuthal profiles showed a degree of orientation for 10% MG samples and 5% MG samples. Generally, these observations agree with the orientation analysis performed by using optical microscopy (Figure 2). It is however surprising that for the reference samples, the azimuthal intensity profile (Figure 4B) also shows orientation, while no such orientation was observed in the optical microscopy images. This might be related to the small scan area, especially used for the reference samples, in that both reference samples show orientation locally which would disappear in a larger scan. For the sonicated 10% MG sample, the preferential orientation is toward 0° (corresponding to the horizontal (*X*-axis) direction in Figures 5 and S4). Meaning that the plane normal of the lipid-crystal platelets is oriented in the horizontal (*X*-axis) direction in Figures 5 and S4, or that the planes of the platelets are vertical (*Y*-axis). In contrast to this, the reference sample shows preferential orientation toward 40°. 5% MG sonicated in turn showed orientation toward 80°, partially obstructed by the detector gaps. While the 5% MG reference showed orientation toward 0°. All azimuthal plots show regular throughs at approximately 25° intervals; this is an artifact produced by the detector used in the measurements.

**Figure 5:**
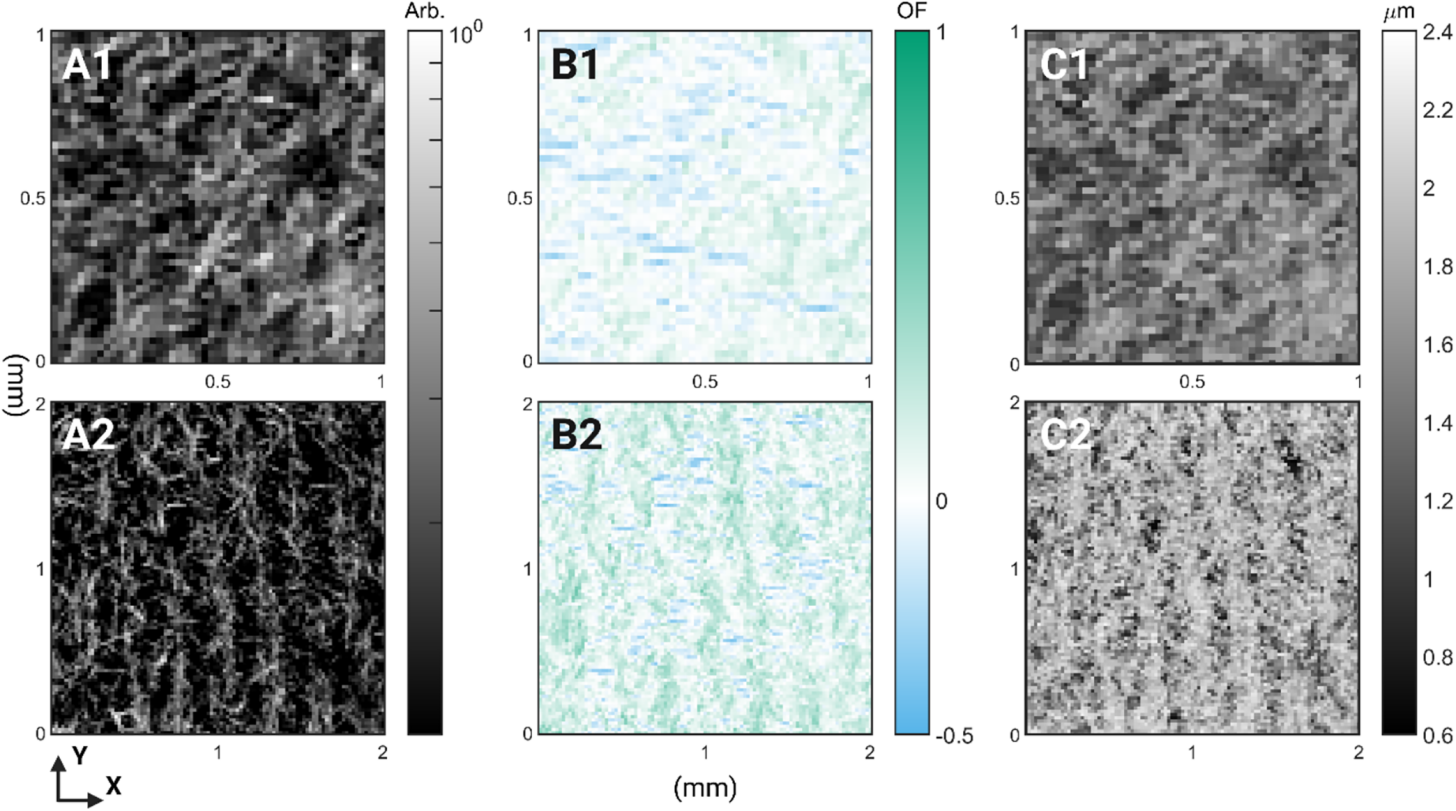
(A) Intensity maps at *q* = 0.13 Å^–1^ for (1) reference and (2) sonicated 10% MG samples. Color scale is logarithmic. (B) Orientation factor (OF) maps at *q* = 0.13 Å^–1^ for (1) reference and (2) sonicated 10% MG samples. (C) Correlation length maps (nm) for (1) reference and (2) sonicated 10% MG samples. For the images of the sonicated samples, the position of the actuating transducer was such that the face of the transducer was pointing approximately horizontally (*X*-axis) to the images. *X*- and *Y*-axes for the maps indicate the scan axis in mm.

To better understand the variations between the samples, the location and full width at half-maximum (fwhm) of the main diffraction peaks in the radial plot were determined for all samples (Table 1). The full width at half-maximum (fwhm) of the main diffraction peak is related to the correlation length of the formed crystals in inverse proportion. The fwhm, along with the obtained uncertainty, is reported in Table 1, second column. 10% MG oleogels showed a smaller fwhm in comparison with 5% samples, with corresponding mean correlation lengths of 1440 ± 510 and 1770 ± 590 nm (Table 1, third column) nm for reference- and sonicated 10% MG samples, respectively. For 5% MG samples, there was no change in the fwhm between reference and sonicated samples, wherein both showed a correlation length of approximately 960 ± 130 nm. The overall distribution of the correlation length for the scanned areas is shown in Figure S2. For the 5% MG oleogels, no meaningful change in the distribution of correlation lengths could be observed. While for the 10% MG samples, a radical change in the distribution shape was evident; 10% MG sonicated sample showed a distribution skewed to larger correlation lengths. This radical shift is also evident in the median value of the fwhm for 10% MG, corresponding to correlation lengths of 1450 ± 220 and 1830 ± 240 nm for reference and sonicated samples, respectively. The observed values for the correlation length are in line with the lipid-crystal platelet thicknesses observed via X-ray microtomography.^29^ Finally, the position of the main diffraction peak was evaluated (Table 1, fourth column). No meaningful change could be observed with all samples showing a peak position of 0.1308 Å^–1^, corresponding to a lamellar thickness of 4.8 nm.

**Table 1:** Derived Values from Peak-Finding and Power-Law Model Fitting^a^

sample	mean Δ*q* (Å^–1^)	median Δ*q* (Å^–1^)	correlation length (nm)	position of peak (Å^–1^)	exponent (|*P*|)
10% MG ref	0.0045 ± 0.001	0.0043 ± 0.001	1440 ± 510	0.1307 ± 0.0003	3.51 ± 0.14
10% MG son	0.0037 ± 0.001	0.0034 ± 0.001	1770 ± 590	0.1308 ± 0.0003	3.58 ± 0.08
5% MG ref	0.0067 ± 0.001	0.0066 ± 0.001	960 ± 130	0.1308 ± 0.0003	3.38 ± 0.07
5% MG son	0.0067 ± 0.001	0.0067 ± 0.001	960 ± 130	0.1310 ± 0.0003	3.57 ± 0.07

a Uncertainty is reported as 2-sigma, except for the fifth column, which is reported as median absolute deviation.

The observed reduction in the fwhm could be interpreted as a 23% increase in crystal thickness on average for 10% MG sonicated sample or a 26% increase in the median thickness produced by the change in correlation thickness distributions between the 10% MG reference and sonicated samples (Figure S2). In contrast to this, 5% MG did not show changes in the fwhm of the main diffraction peak, and as such, no changes in neither the distribution nor average values of crystal thickness were observed. However, the interpretation of the fwhm being proportional to the inverse of the platelet thickness might not be entirely suitable. This is because the utilized X-ray beam had dimensions of 17 μm × 23 μm, effectively integrating the platelet properties over an area of around 400 μm^2^ and over the full thickness of the measured sample. As such, the methodology is incapable of distinguishing between single thicker platelets, and thinner platelets in alignment with each other (i.e., stacked) inside the beam area, as would be the case for micrometer-thickness MG crystalline platelets oriented by the acoustic radiation force (USW). This would be a simpler explanation to the observed increase in correlation length, and seemingly supported by the observations made with optical microscopy, which showed orientation of platelets for 10% MG sonicated samples, but no orientation for 5% MG samples.

SAXS scans were fitted with a power-law model (eq 1) between *q* values of 0.005 and 0.05 Å^–1^ (power-law region). Which correspond to length scales between 125.7 and 12.6 nm. This length scale corresponds to MG nanoplatelets formed from the lamellae.^30^ The power-law model contains an exponent *P*.^21,22^ This exponent contains information on the surface topography that produced the scattering. Additionally, a surface fractal dimension *D*_s_ can be derived from the exponent *P*.^21,31,32^ In Table 1, fifth column, the values for the exponent |*P*| obtained from power-law fitting on averaged radial data (Figure 4A) are observed. All obtained exponent values fall between 3 and 4. The derived exponents for the samples are not statistically different from each other. The only notable difference is the 10% MG reference, which has a slightly lower exponent |*P*| with a higher variance in the fit. The corresponding *D*_s_ values are between 2.42 and 2.62, indicating some roughness in the existing surfaces or boundaries. To obtain a deeper understanding on the surface morphology of the samples, the power-law fitting was repeated for all obtained data points separately. The results (values for |*P*|) were then plotted as histograms (Figure S3). The observed distributions show skewed Gaussian behavior for 10% MG, and 5% MG sonicated samples, while the 5% MG reference shows no skewness. Based on these distributions, especially the 5% MG sample showed an increase in the exponent |*P*| due to sonication.

The results of the power-law fitting showed that out of the four samples measured, 10% MG sonicated had the smoothest morphology, followed by 5% MG sonicated, 10% MG reference, and finally 5% MG reference. This was in line with the trend observed in the correlation length for the same samples. That is, larger concentrations of MG resulted in smoother nanoplatelet surfaces or boundaries. The effect of USW was also to decrease the surface roughness. The increase in smoothness was, though small, statistically significant as clearly observed for 5% MG samples (Figure S3) and by a *t* test for 10% MG as detailed in the Supporting Information. However, results from the power-law model fitting are difficult to corroborate without cryogenic transmission electron microscopy (Cryo-TEM) or by simulations, and as such, the reasoning for the apparent increase in smoothness remains unclear.

The aim of USW treatment is to generate MG crystal structures that are beneficial in specific use cases. While changes, on average, are still informative, we are more focused on local changes to the density and orientation of MG platelets due to sonication. For this purpose, SAXS scans of samples were carried out with a scanned area of 2 × 2 mm for sonicated samples and 1 × 1 mm for reference samples. The results for 10% MG samples are displayed in Figure 5. In Figure 5, the first column shows the intensity of the peak at *q* = 0.13 Å^–1^, corresponding to the main diffraction peak from the MG lamellae (Figure 4A). In the second column of Figure 5, the local OF is calculated for the diffraction Debye–Scherrer ring at *q* = 0.13 Å^–1^. As such, the plotted data in the second column inform us about the local orientation of the MG lamellae that comprise the larger crystal platelets. A OF value of −0.5 denotes orientation of the plane normal perpendicular to the director, and a value of 1 is parallel to the director. In these figures, the director is in alignment with the horizontal (*X*-axis) direction of Figures 5 and S4. This means that OF values observed in the vertical bands (i.e., values close to 1) indicate that the plane normal of the lipid crystals in these regions is facing in the horizontal (*X*-axis) direction; i.e., the planes of the lipid crystals are oriented along the observed vertical bands of denser crystalline material. Finally, in the third column of Figure 5, the local distribution of the calculated correlation lengths is shown.

The intensity map for the reference 10% MG (Figure 5A1) shows an inhomogeneous distribution of the crystalline material, with the area closer to the bottom-right corner containing denser regions of material. This is in line with other measurements performed (optical microscopy and CESAM), which also showed reference samples containing an inhomogeneous distribution of crystalline material. For the sonicated 10% MG sample, a periodic band-like structure of denser crystalline material is observable. The spacing of the observed bands was approximately 0.3 mm. For 10% MG intensity maps, the outline of singular crystalline platelets was visible, especially for the sonicated sample. The orientation of the lamellae for the reference 10% (Figure 5B1) shows regions of both negative and positive OF values, along with regions where no preferential orientation is observed (values close to zero). Regions with an OF value of zero would seem to correlate with regions of low diffracted intensity (cf. Figure 5A with Figure 5B). Light blue colored regions would seem to correlate with clearly visible singular crystals in the intensity maps, which would seem to be oriented in the horizontal (Figures 5 and S4 *X*-axis) direction. Green regions (oriented toward the director) form a more continuous mass in the reference image but are clearly more intense for the image of the sonicated sample, along with clustering to the observed bands in the intensity map of the sonicated sample. This would indicate that platelets in the band-like regions are oriented parallel with the bands toward the director (horizontal direction or *X*-axis, Figures 5 and S4 *X*-axis). MG platelet thickness (as approximated by the correlation length) also seems to correlate with the density of crystalline material (cf., Figure 5A,5C). Especially noteworthy would be that for the 10% MG sonicated sample, larger correlation lengths (thickness) are observed to follow the band structure seen in the intensity maps.

Figure 5C1,C2 shows that thicker platelets are localized at regions of high concentration, which for the sonicated sample is the nodal planes as discussed before. Some experiments with crystallizing wax have shown an effect in the nucleation and crystal growth produced by low-intensity ultrasound.^33^ As such, another explanation for the observed increase in correlation length could be a yet-to-be-explained effect purely on the basis of the acoustic pressure. However, the discrepancy that no increase in correlation length was observed for USW-treated 5% MG oleogels (Table 1) would seem to discredit this hypothesis. A third explanation for the increase could be a local concentration increase produced by the acoustic radiation force, wherein the crystal growth of platelets is affected by the local increase in the concentration of crystalline material. The effect of concentration on MG platelet properties was apparent from the results obtained here (cf., 10% MG reference and 5% MG reference; Table 1).

A surprising observation is that for the whole scanned area, a preferred orientation could be seen in the case of 10% MG for both reference and sonicated samples (Figure 4B, cf. dotted lines with solid lines). This seemed to contradict optical microscopy, which clearly indicated no preferred orientation for the 10% MG reference. The local intensity- and OF maps (Figure 5A1,B1) do seem to provide an explanation: the outlines of single platelets are clearly visible. It is possible to assess from the intensity map that the platelets are mostly slanted toward the upper right corner of the figure (i.e., approximately 50°). The OF map confirms this, wherein the green regions (corresponding to platelets in the intensity maps) indicate an orientation toward the director (horizontal direction, Figures 5 and S4 *X*-axis). However, the scans for the 10% MG reference sample show no structure in the orientation. It is as such possible and even probable due to the small scan area (1 × 1 mm) that the imaged scan area for the 10% MG reference sample was an outlier, wherein platelets had formed with preferred orientation due to happenstance. It is expected that, had the scan area been larger, no such preferred orientation would have been observed. In contrast to this, the 10% MG sonicated samples show that the preferred orientation is localized to the band-like regions of denser crystalline material. Inside these regions, the orientation is perfectly parallel with the observed bands, as would be expected had this orientation been produced by the acoustic radiation force in a standing wave field.

The same analysis for local properties was carried out for 5% MG samples (Figure S4). Due to signal-to-noise issues, it is more difficult to ascertain similar observations in comparison with 10% MG samples.

## 4 Conclusions

In this work, the effect of ultrasound standing wave-induced acoustophoresis on the dynamically forming lipid-crystal network of monoglyceride oleogels was investigated utilizing multiple complementary imaging and characterization techniques, for which findings are presented in Table 2.

**Table 2:** Compilation of Observations Found Utilizing the Measurement Methods Presented

method	contrast mechanism	finding
optical microscopy	visible-spectrum light scattering from contrasting refractive index	USW causes localization of MG platelets into acoustic nodal planes; USW causes orientation of MG platelets
CESAM	wide-bandwidth high-frequency acoustic wave scattering from contrasting mechanical impedance	USW produces band-like regions of denser and less dense MG crystalline material
high-*q* range X-ray scattering	X-ray scattering from MG nanocrystal lamellar structure	confirms optical microscopy and CESAM findings; in addition, USW treatment shows an increase in correlation length, which might indicate increase in platelet thickness
low-*q* range X-ray scattering	X-ray scattering from MG platelet surface	USW produced rougher nanoplatelet surfaces, as observed from power-law fitting; this trend was in line with observations on the correlation length

USW showed the capability to structure and orient MG platelets undergoing lipid-crystal network formation, as observed utilizing optical microscopy and CESAM. These results were consistent with previous findings which showed localization of crystalline material into band-like structures when subjected to USW treatment. Analysis of the high-*q* SAXS pattern results showed a possible increase in the MG platelet thickness as a result of USW application for 10% MG oleogels. However, this finding was not seen in the 5% MG oleogel sample. As such, the reason why and how this possible increase in platelet thickness occurred are difficult to ascertain. In correlation with these results, SAXS data showed an increase in the nanoplatelet surface smoothness as a result of USW treatment, which was observed for both 10 and 5% MG oleogels. As such, a possible transition on the nanoscopic level (increase in correlation length) and on the microscopic level (migration of platelets) was observed due to USW treatment. These results further our understanding of how mechanical changes occur in USW-treated oleogels; while it is presumed that localized density differences provide most of the mechanical change, these results indicate that lipid-crystal orientation and possible platelet thickness differences might also provide contributions.

## Supplementary Material



## Notes

Raw example SAXS images showing Debye−Scherrer rings; histograms showing the distributions of correlation lengths; power-law exponent values over the scanned SAXS area; and local SAXS intensity maps, orientation factor, and correlation length maps for 5% MG sonicated and reference (PDF)
