# Correction to “Effect of Ultrasound Standing Wave-Induced Acoustophoresis in Monoglyceride Oleogel Structuration”

**DOI:** 10.1021/acs.cgd.5c01579

**Published:** 2025-12-09

**Authors:** Petri Lassila, Thomas Zinn, Jere Hyvönen, Enriqueta Noriega Benitez, Paavo Penttilä, Ari Salmi, Fabio Valoppi

In the original publication, in section 3.3, although the correlation lengths were reported in nanometers (nm), the numerical values corresponded to angstroms (Å). As a result, the reported values were 10 times larger than intended.

On page 4400, in the third paragraph of section 3.3, sentence 4 should be corrected as follows:

“10% MG oleogels showed a smaller fwhm in comparison with 5% samples, with corresponding mean correlation lengths of 144 ± 51 and 177 ± 59 nm (Table 1, third column) for reference- and sonicated 10% MG samples, respectively. For 5% MG samples, there was no change in the fwhm between reference and sonicated samples, wherein both showed a correlation length of approximately 96 ± 13 nm.”

Sentence 9 of the same paragraph should be corrected as follows:

“This radical shift is also evident in the median value of the fwhm for 10% MG, corresponding to correlation lengths of 145 ± 22 and 183 ± 24 nm for reference and sonicated samples, respectively.”

Corrected Table 1 and Figure 5C are shown herein. The Supporting Information has also been corrected.

**Table 1:** Derived Values from Peak-Finding, and Power-Law Model Fitting^a^

sample	mean Δ*q* (Å^–1^)	median Δ*q* (Å^–1^)	correlation length (nm)	position of peak (Å^–1^)	exponent (|*P*|)
10% MG ref	0.0045 ± 0.001	0.0043 ± 0.001	144 ± 51	0.1307 ± 0.0003	3.51 ± 0.14
10% MG son	0.0037 ± 0.001	0.0034 ± 0.001	177 ± 59	0.1308 ± 0.0003	3.58 ± 0.08
5% MG ref	0.0067 ± 0.001	0.0066 ± 0.001	96 ± 13	0.1308 ± 0.0003	3.38 ± 0.07
5% MG son	0.0067 ± 0.001	0.0067 ± 0.001	96 ± 13	0.1310 ± 0.0003	3.57 ± 0.07

a Uncertainty is reported as 2-sigma, except for fifth column, which is reported as median absolute deviation.

**Figure 5:**
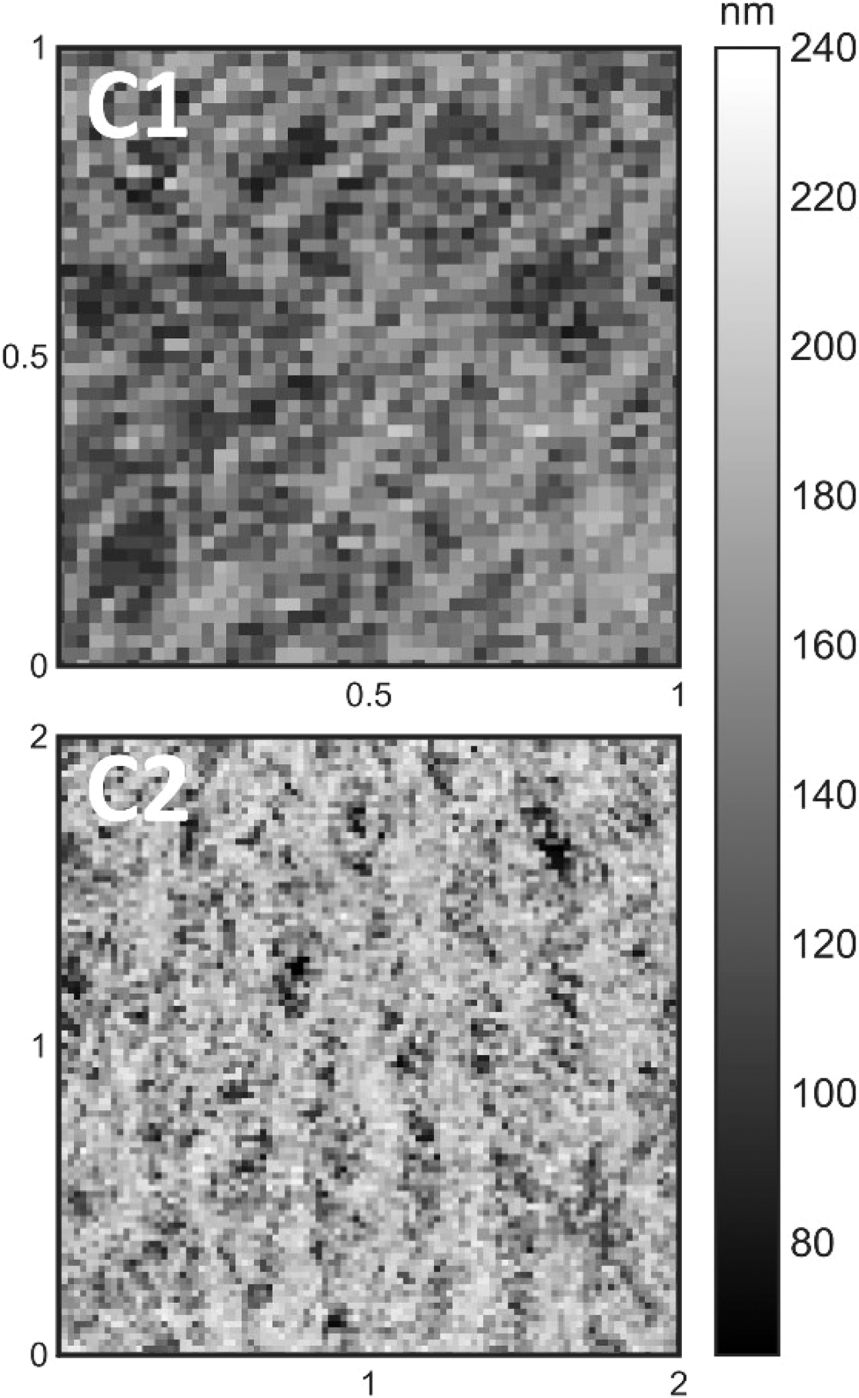
(C) Correlation length maps (nm) for (1) reference and (2) sonicated 10% MG samples.

## Supplementary Material



## Notes

Raw example SAXS images showing Debye–Scherrer rings; histograms showing the distributions of correlation lengths; power-law exponent values over the scanned SAXS area; and local SAXS intensity maps, orientation factor, and correlation length maps for 5% MG sonicated and reference (corrected) (PDF)

