# Supplementary material for: Effect of Ultrasound Standing Wave-Induced Acoustophoresis in Monoglyceride Oleogel Structuration
Source: Cryst Growth Des. 2025 Jun 3;25(12):4394–404. doi: 10.1021/acs.cgd.5c00291 (PMC12186261; doi:10.1021/acs.cgd.5c00291)
Supplement: Supplementary file 1 [file cg5c00291_si_001.pdf]

SUPPLEMENTARY INFORMATION

# The effect of ultrasound-standing-wave induced acoustophoresis in monoglyceride oleogel structuration

Petri Lassila[1], Thomas Zinn [2], Jere Hyvönen[1], Enriqueta Noriega Benitez[3], Paavo Penttilä[3], Ari Salmi[1], Fabio Valoppi[1,4,5,6,7]

Affiliations:
[1] Electronics Research Laboratory, Department of Physics, P.O. Box 64 (Gustaf Hällströmin katu 2), FI-00014 University of Helsinki, Finland
[2] Diamond Light Source Ltd., Didcot, Oxfordshire, OX11 ODE, United Kingdom
[3] Department of Bioproducts and Biosystems, School of Chemical Engineering, Aalto University, Vuorimiehentie 1, Espoo 02150, Finland
[4] Department of Food and Nutrition, P.O. Box 66 (Agnes Sjöbergin katu 2), FI-00014 University of Helsinki, Finland
[5] Helsinki Institute of Sustainability Science, Faculty of Agriculture and Forestry, FI-00014 University of Helsinki, Finland
[6] Helsinki Institute of Life Science, University of Helsinki, 00014, Helsinki, Finland
[7] Perfat Technologies, Agnes Sjöbergin katu 2, 00790 Helsinki, Finland

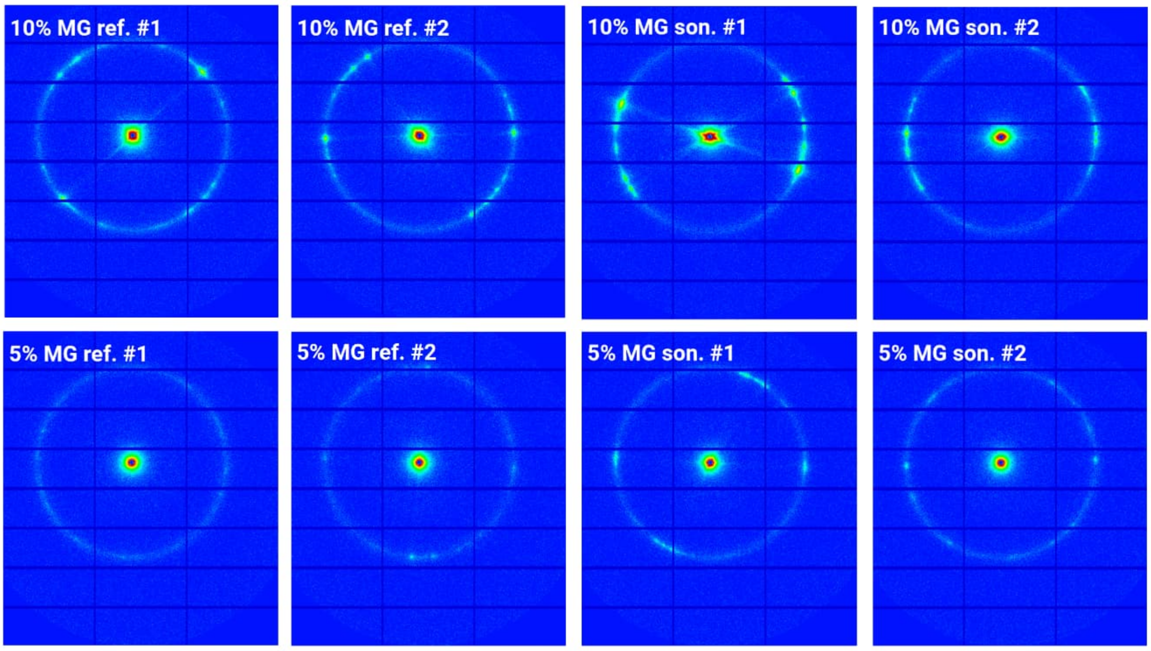


***Figure S1.*** *Example SAXS images of Debye-Scherrer rings for 10% & 5% MG reference and sonicated oleogels.*

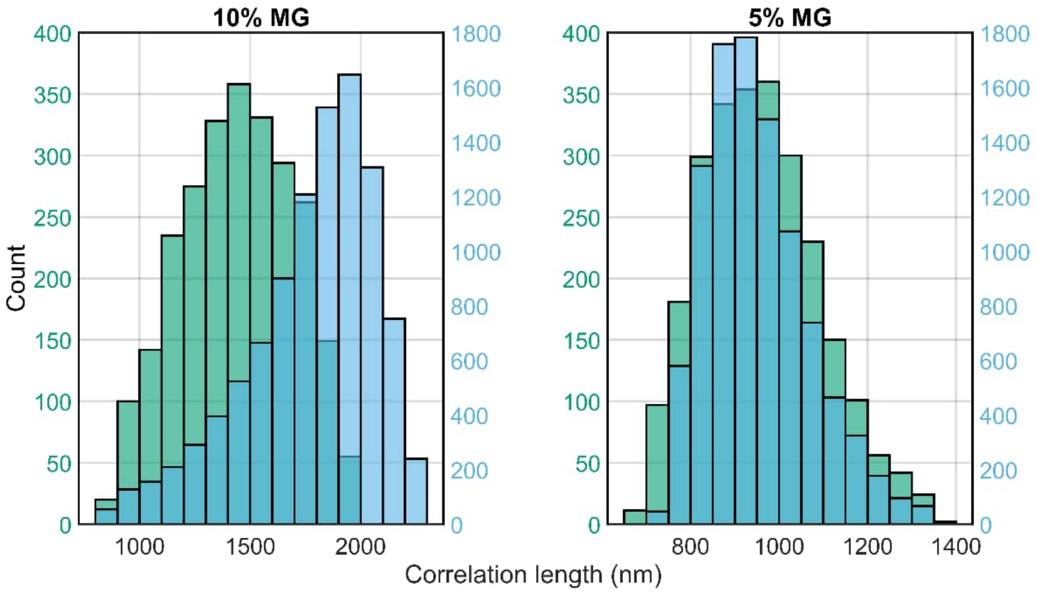


***Figure S2.*** *Histogram for correlation length for 10%- and 5% MG. Y-axis shows the number of counts. Green bars show the distribution for reference oleogels, while the blue bars show the distribution for the sonicated samples.*

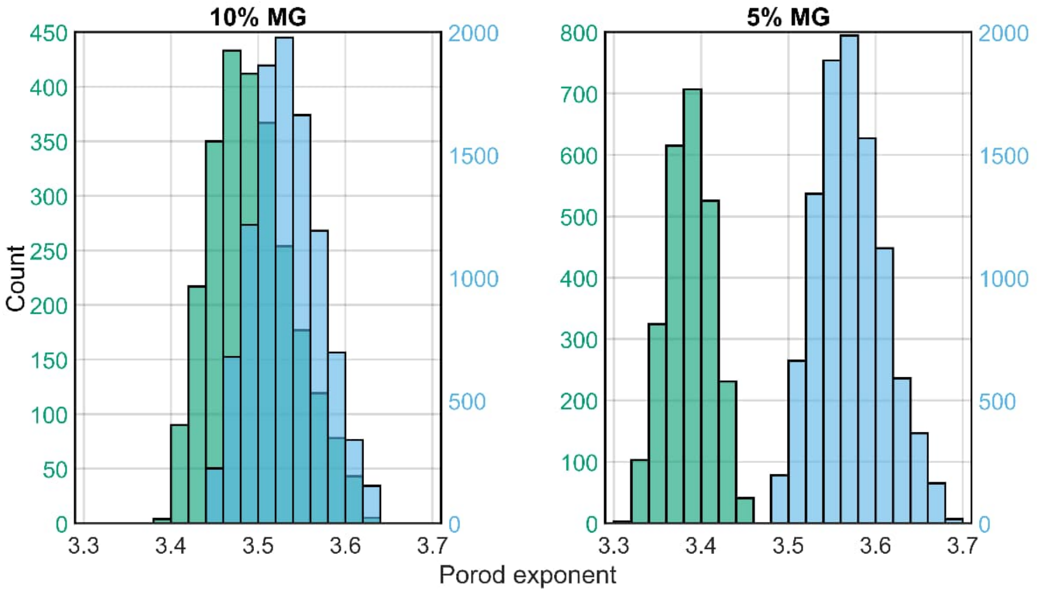


***Figure S3.*** *Histogram of the Porod exponent values (|P|) for 10%- and 5% MG scans. Y-axis shows the number of counts. Green bars show the distribution for reference oleogels, while the blue bars show the distribution for the sonicated samples.*

In figure S3., the distribution of the exponent |P| for all scans is shown. A one-sided, two sample T-test, with no assumption made on the equality of the variances was carried out for 10 wt% MG sample scans. The null-hypothesis being that the |P| distributions come from the same distribution, and the alternate hypothesis that 10 wt% MG reference showed a population mean lower than 10% MG sonicated. The null-hypothesis was rejected with a p-value of zero. That is, scans of sonicated samples showed a higher mean-value for the exponent |P| with respect to scans of reference samples. Wherein sonication showed a 5.6% increase in P for 5 wt% MG, and a 2% increase for 10 wt% MG.

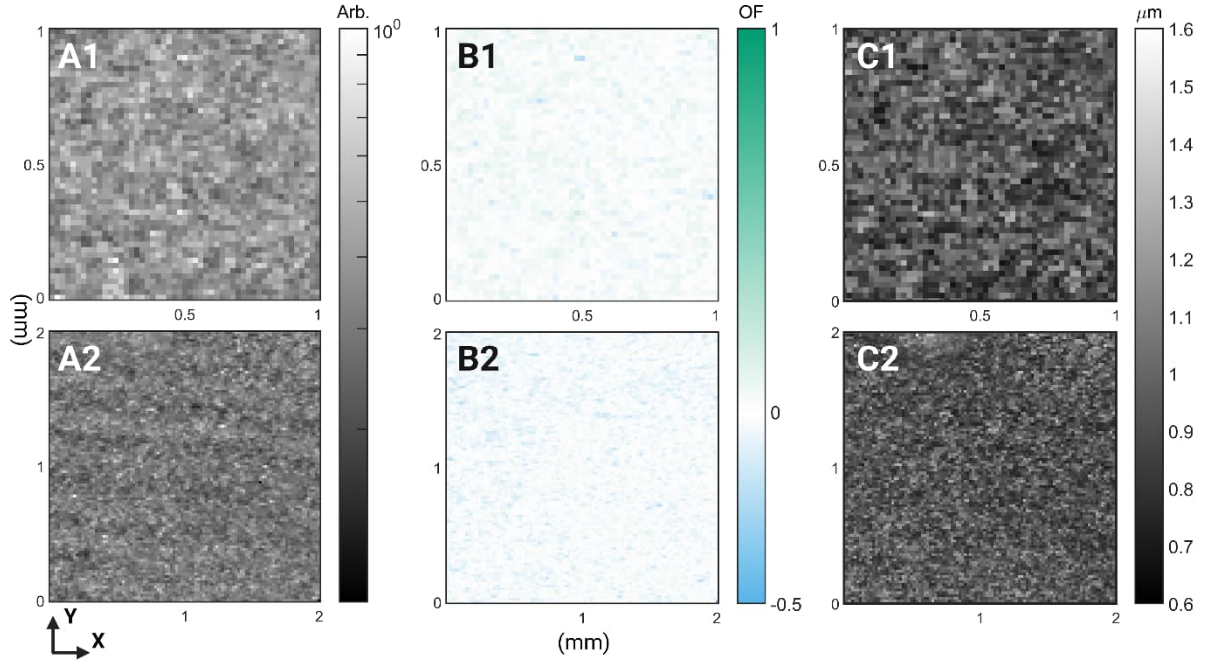


***Figure S4.*** *A) Intensity maps at q = 0.13 Å*$^{-1}$ *for (1) reference and (2) sonicated 5% MG samples. Color scale is logarithmic. B) OF-maps at q = 0.13 Å*$^{-1}$ *for (1) reference and (2)*

*sonicated 5% MG samples. C) Correlation length maps (nm) for (1) reference and (2) sonicated 5% MG samples. For the images of the sonicated samples, the position of the actuating transducer was such that the face of the transducer was pointing approximately vertical to the images. X-, and Y-axis for the maps indicate the scan axis in mm.*

Figure S4A shows the intensity map for reference and sonicated 5% MG samples, fig. S4B the local OF, and fig. S4C the local distribution of correlation lengths. The intensity maps for both reference and sonicated 5% MG oleogels seem to resemble static noise, with no major clumping visible for either sample. For the sonicated sample, an extremely faint outline of the band-structure is however, visible. Unlike in the case of 10% MG, no outlines for singular crystals could be observed for neither reference nor sonicated samples. In the OF map (fig. S4B) no structured preferential orientation is visible. Aside from local regions with small amount of preferred orientation, the OF map once again resembles static noise. The notable result for the correlation length map for 5% MG (fig. S4C), is observed for the sonicated sample: wherein a region in the top-left corner of the map shows a larger area of higher correlation lengths. This (possibly clumping) was not visible in the intensity map for the sonicated sample.
